# Clinical trial reporting performance of thirty UK universities on ClinicalTrials.gov—evaluation of a new tracking tool for the US clinical trial registry

**DOI:** 10.1186/s13063-021-05330-5

**Published:** 2021-06-01

**Authors:** Sarai Mirjam Keestra, Florence Rodgers, Daphne Lenz, Rhiannon Osborne, Till Bruckner, Sean Lee

**Affiliations:** 1 Department of Global Health & Development, London School of Hygiene and Tropical Medicine, London, UK; 2 Amsterdam UMC, University of Amsterdam, Amsterdam, The Netherlands; 3 School of Medicine, Imperial College London, London, UK; 4 Department of Science, Technology and Society, University of Vienna, Vienna, Austria; 5 School of Clinical Medicine, University of Cambridge, Cambridge, UK; 6 BIH QUEST Center, Berlin, Germany; 7 TranspariMED, Bristol, UK; 8 Market Securities LLP, London, UK

**Keywords:** Clinical trials, Transparency, Publication bias, Research waste, ClinicalTrials.gov, Tracking tool, FDAAA 2007

## Abstract

Clinical trial transparency forms the foundation of evidence-based medicine, and trial sponsors, especially publicly funded institutions such as universities, have an ethical and scientific responsibility to make the results of clinical trials publicly available in a timely fashion. We assessed whether the thirty UK universities receiving the most Medical Research Council funding in 2017–2018 complied with World Health Organization best practices for clinical trial reporting on the US Clinical Trial Registry (ClinicalTrials.gov). Firstly, we developed and evaluated a novel automated tracking tool (clinical-trials-tracker.com) for clinical trials registered on ClinicalTrials.gov. This tracker identifies the number of due trials (whose completion lies more than 395 days in the past) that have not reported results on the registry and can now be used for all sponsors. Secondly, we used the tracker to determine the number of due clinical trials sponsored by the selected UK universities in October 2020. Thirdly, using the FDAAA Trials Tracker, we identified trials sponsored by these universities that are not complying with reporting requirements under the Food and Drug Administration Amendments Act 2007. Finally, we quantified the average and median number of days between primary completion date and results posting. In October 2020, the universities included in our study were sponsoring 1634 due trials, only 1.6% (*n* = 26) of which had reported results within a year of completion. 89.8% (*n* = 1468) of trials remained unreported, and 8.6% (*n* = 140) of trials reported results late. We also identified 687 trials that contained inconsistent data, suggesting that UK universities often fail to update their data adequately on ClinicalTrials.gov. The mean reporting delay after primary completion for trials that posted results was 981 days, the median 728 days. Only four trials by UK universities violated the FDAAA 2007. We suggest a number of reasons for the poor reporting performance of UK universities on ClinicalTrials.gov: (i) efforts to improve clinical trial reporting in the UK have to date focused on the European clinical trial registry (EU CTR), (ii) the absence of a tracking tool for timely reporting on ClinicalTrials.gov has limited the visibility of institutions’ reporting performance on the US registry and (iii) there is currently a lack of repercussions for UK sponsors who fail to report results on ClinicalTrials.gov which should be addressed in the future.

**Supplementary Information:**

The online version contains supplementary material available at 10.1186/s13063-021-05330-5.

## Introduction

‘Do no harm’ is a fundamental principle governing medical practice. In order to provide the best care for patients, clinicians and healthcare guideline providers must have the necessary information to make evidence-based decisions. Clinical trial sponsors therefore have an ethical and scientific obligation to provide complete information about the efficacy and safety of health technologies. The World Health Organization (WHO) recommends that trial sponsors make the key outcomes of all clinical trials available on the registry where they were originally registered within 12 months of study completion [1]. However, despite global efforts to combat delayed or incomplete reporting of clinical trials, results often remain unpublished, particularly if ‘negative’ or not statistically significant [2]. The COVID-19 pandemic has further stimulated calls for ‘radical transparency’ and brought the attention of the global scientific community to the issue of transparent and timely reporting of trial results [3]. Notably, clinical trial registries offer the opportunity to share results faster and, in more detail, than peer-reviewed journals do [4].

On 29 July 2020, the NHS Health and Research Authority released a new strategy to promote transparency and openness in health and social care research in the UK, in which it highlighted that 30% of clinical trials (excluding trials of investigative medicinal products) were not being registered and up to 25% of results of clinical trials of medicines were not being reported [5, 6]. In 2019–2020, efforts were made by the House of Commons’ Science and Technology Committee to improve clinical trial reporting by NHS trusts and universities, focusing on the EU Clinical Trials Register (EU CTR) [7]. However, the EU CTR does not allow registration of all trial types, so UK universities often use ClinicalTrials.gov to register certain types of clinical trials, including trials of medical devices. The 2007 Food and Drug Administration Amendments Act legally requires certain trials involving FDA-regulated drugs and medical devices to post summary results within 12 months of completion, and the FDA seems to be preparing to impose financial penalties for non-compliance [8–10]. The US law only applies to a small minority of trials sponsored by UK universities that are registered on the US registry. However, by registering trials on ClinicalTrials.gov, UK universities have an ethical and scientific obligation to post the results of those trials on the registry and keep their registry entries up to date, as set out in WHO best practices [1]. Indeed, article 36 of the *Helsinki Declaration on Ethical Research Involving Human Subjects* also states that “researchers have a duty to make publicly available the results of their research on human subjects and are accountable for the completeness and accuracy of their reports” [11]. Evidence suggests that results uploaded on such clinical trial registries are often more extensive and complete than later publications [12]. In addition, registry reporting accelerates medical progress by enabling researchers to share their results rapidly, in advance of publication in a peer reviewed journal. Even in the absence of legal requirements, scientific best practice as defined by the WHO requires the pre-registration of all trials and the periodic updating of registry data. The WHO Statement on public disclosure of clinical trial results specifies that 12 months is “the longest possible acceptable timeframe for reporting and shorter timeframes are strongly encouraged” [1].

Here, we aimed to develop and verify a tracking tool that, in contrast to existing trackers, identifies missing trial results on ClinicalTrials.gov regardless of legal status and thus measures trial sponsors’ adherence to global best practices rather than their narrow legal compliance. Our tracker allows all trial sponsors with interventional trials registered on ClinicalTrials.gov to easily identify trials that have not yet been fully reported. Additionally, we aimed to create an overview of unreported trial data on ClinicalTrials.gov for the top Medical Research Council (MRC) funded UK universities and identify trials sponsored by those universities that are violating the FDAAA 2007.

## Methods

**Fig. 1:**
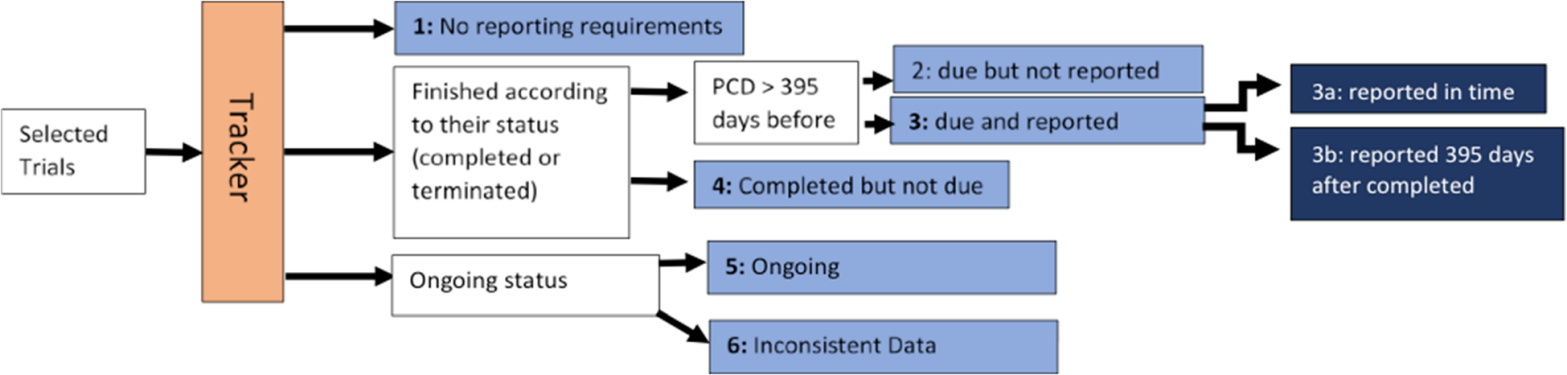
A visual overview of the tracker output from the novel US clinical-trials-tracker

We developed a novel tracking tool, which identifies the number of interventional clinical trials on ClinicalTrials.gov with a primary completion date more than 395 days in the past that have not posted summary results in tabular format on the registry. The 395-day cut-off point used to identify due trials includes a 30-day grace period to allow for review of submitted data by ClinicalTrials.gov staff after the sponsor has uploaded results. Within the due trials, we differentiated between those that remained unreported on the registry, due trials that were reported late, i.e. after the 395 days cut-off, and due trials that were reported on time, i.e. within 395 days of the primary completion date (Fig. 1). Further, we used the tracker to compare trial status with the primary completion date to identify trials that may be incorrectly listed as ongoing, trials with an unknown status, and ongoing trials lacking a primary completion date, which we classified as inconsistent. The novel tracking tool has the following categories, based on which the percentage of due clinical trials that have not reported their results on ClinicalTrials.gov in a timely fashion can be calculated.

1. **No reporting requirement**
  All trials that are listed as ’Suspended’ or ’Withdrawn’ are not required to report results. They are sorted by the tracker to this category even if there is inconsistent data (e.g. no primary completion date) or if they have reported results (these results are ignored).
2. **Due but not reported**
  Trials that have finished (’Completed’ or ’Terminated’) that have not posted results yet and have exceeded the reporting timeframe. Trials that have finished that have not posted results and have no primary completion dates are also classed under this category.
3. **Due and reported**
  Trials that have finished (’Completed’ or ’Terminated’) that have reported their results. Late posting of results will also be in this category.
  - *Due and reported late*
    Trials that have finished (’Completed’ or ’Terminated’) and have reported their results more than 395 days after their primary completion date.
  - *Due and reported in time*
    Trials that have finished (’Completed’ or ’Terminated’) and have reported their results within 395 days.
4. **Completed/terminated but not due**
  - *Results not due yet*
    Trials that have finished (’Completed’ or ’Terminated’) less than 395 days ago that have not posted results yet.
  - *Results not yet expected but have reported*
    Trials that have finished (’Completed’ or ’Terminated’) less than 395 days ago but have reported results.
5. **Ongoing**
  Trials that are ongoing with primary completion date in the future and have not posted results.
6. **Inconsistent data**
  Trials that are:
  ◦ Marked as having an 'unknown status'.
  ◦ Trials that have a completion date in the past but remain marked as ‘ongoing'
    ▪ These are trials that should have already been completed but are self-declaring as ongoing, this is contradictory and therefore inconsistent.
  ◦ Ongoing trials that lack a completion date
    ▪ Trials should have an expected primary completion date. Not having this is an inconsistency.

The accuracy of the tracker was manually validated; the validation methodology and results can be found in Supplementary File 1. The Github code for our tracker has been made available freely online (https://github.com/LeeSean96/GlobalHealthRanking) and updated results from the tracker will be posted on the website clinical-trials-tracker.com as a csv file on a monthly basis alongside a user-friendly interface that is currently undergoing testing and further development.

Using this novel tracking tool, we assessed the compliance of UK universities with WHO best practices on results reporting on the US Clinical Trial Registry (ClinicalTrials.gov) in October 2020. The thirty universities receiving the largest total 2017-2018 Medical Research Council (MRC) research grants were selected for analysis [13], which is the latest year for which this information was available. The tracker also quantifies the number of days between the primary completion date and the date that results were published on the ClinicalTrials.gov registry, based on which we calculated the mean and median time delay for those trials reporting results. Additionally, we utilised the FDAAA Trials Tracker developed by the Evidence-Based Medicine DataLab at the University of Oxford to identify any trials sponsored by these UK universities that are not complying with US disclosure law (http://fdaaa.trialstracker.net/). We downloaded the data from ClinicalTrials.gov using our novel tracking tool on 19 October 2020 and used the FDAAA Trials Tracker on 28 October 2020.

## Results

**Fig. 2:**
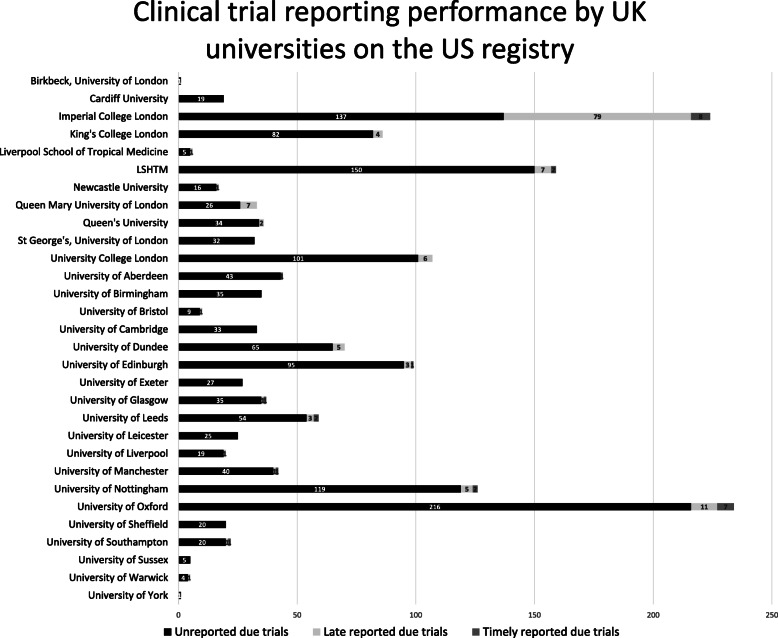
Clinical trial reporting performance of thirty UK universities on the US clinical trial registry (ClinicalTrials.gov), differentiated between timely, late and unreported trials, as of October 2020

In October 2020, the thirty UK universities included in our study were listed as being the lead sponsors of 3034 trials on ClinicalTrials.gov. The University of Oxford and Imperial College London had the most trials registered on the US registry, 425 and 390 respectively. Eleven universities had fewer than fifty trials registered on ClinicalTrials.gov (Supplementary File 2 shows results per university). Of all trials sponsored by the thirty UK universities included in the study cohort, 1634 were completed and had a primary completion date more than 395 days in the past. Of these due trials, 26 (1.6%) had reported results in a timely manner on ClinicalTrials.gov, adhering to the WHO’s best practice timeframe of 12 months (Table 1). One hundred forty trials (8.6%) reported results later than 395 days after the primary completion date. One thousand four hundred sixty-eight trials (89.8%) did not report tabular summary results on the registry (Fig. 2). Furthermore, we found 687 trials (42.0%) containing inconsistent data.

**Table 1:** Overview of due trials sponsored by thirty UK universities on the ClinicalTrials.gov, sorted according to whether they reported within the 395 day cut-off, reported late, or did not report at all, as of October 2020. We calculated the percentage of unreported trials per university by dividing the number of unreported due trials through the total number of due trials

Lead sponsor	Due trials	Timely reported	Late (> 395 days) reported	Unreported	Unreported of all due trials (%)
Birkbeck, University of London	1			1	100.0
Cardiff University	19			19	100.0
Imperial College London	224	8	79	137	61.2
King's College London	86		4	82	95.3
Liverpool School of Tropical Medicine	6		1	5	83.3
London School of Hygiene and Tropical Medicine	159	2	7	150	94.3
Newcastle University	17		1	16	94.1
Queen Mary University of London	33		7	26	78.8
Queen's University, Belfast	36		2	34	94.4
St George's, University of London	32			32	100.0
University College London	107		6	101	94.4
University of Aberdeen	44	1		43	97.7
University of Birmingham	35			35	100.0
University of Bristol	10		1	9	90.0
University of Cambridge	33			33	100.0
University of Dundee	70		5	65	92.9
University of Edinburgh	99	1	3	95	96.0
University of Exeter	27			27	100.0
University of Glasgow	37	1	1	35	94.6
University of Leeds	59	2	3	54	91.5
University of Leicester	25			25	100.0
University of Liverpool	20		1	19	95.0
University of Manchester	42	1	1	40	95.2
University of Nottingham	126	2	5	119	94.4
University of Oxford	234	7	11	216	92.3
University of Sheffield	20			20	100.0
University of Southampton	22	1	1	20	90.9
University of Sussex	5			5	100.0
University of Warwick	5		1	4	80.0
University of York	1			1	100.0
**Total of all 30 Universities**	1634	26	140	1468	89.8

The mean reporting delay after primary completion of the trials was 981 days (median 728 days) for the clinical trials that did report results. The fastest reporting trial was one by the University of Aberdeen, which reported only 22 days after primary completion date (NCT01245270). The longest delay in reporting was for a trial at Imperial College London (NCT00390949) that reported 5943 days after primary completion.

Only four trials sponsored by UK universities in our study were in violation of US law FDAAA as of October 2020. These included one trial reported late by Imperial College London (NCT03380572) and three studies that remain unreported, sponsored by Imperial College London (NCT04355156), University of Liverpool (NCT03323229) and the University of Aberdeen (NCT03770442).

## Discussion

As of October 2020, 1468 trials with a primary completion date more than 395 days in the past that were sponsored by the top thirty MRC-funded UK universities remain without tabular summary results on ClinicalTrials.gov. There were significant delays to reporting beyond the 395-day deadline, with a mean of 981 days between trial completion and results posting. Only 26 (1.6%) trials included in the study reported results in line with WHO best practices. However, only 4 (0.2%) of the due trials in the cohort are subject to FDAAA legal disclosure requirements; neither European Union regulations nor UK national legislation require summary result posting for the other 1464 trials for which results were unreported (89.6%) on ClinicalTrials.gov. Our study therefore suggests the existence of a large gap between legal and regulatory reporting requirements and WHO best practices, and weak institutional adherence to the latter [1].

Although only four trials sponsored by our cohort of UK universities violated FDAAA law in October 2020, it should be noted that at the time the FDAAA 2007 tracker was developed, the law applied to trials conducted after 2017 only. However, following a Federal Court ruling in February 2020, FDAAA now applies to trials conducted between 2007 and 2017 as well [14], something which is not currently reflected in FDAAA tracker data. It is therefore likely that our analysis grossly underestimates the extent to which UK universities are violating legal clinical trial reporting requirements. This is especially concerning as the FDA may fine non-compliant sponsors up to $10,000 for “all violations adjudicated in a single proceeding” and $11,569 for each day that a sponsor fails to report results after the initial 30-day grace period [9]. Although the FDA has so far failed to levy these fines, this appears to be changing, and UK universities may face significant financial penalties in the future.

Our study highlights a gap between the registry reporting performance of UK universities on EU CTR and ClinicalTrials.gov. Due to public, parliamentary, and media attention, the existence of applicable European regulatory guidelines, and the availability of performance data through the EU Trials Tracker, the UK academic sector has made very strong progress on improving results reporting on EU CTR since late 2018 [15, 16]. Our study suggests that UK universities have generally not yet extended their registry reporting efforts to interventional trials listed on ClinicalTrials.gov, at least not retrospectively, despite past trials being of great scientific and clinical value as they often involve medical products already on the market. Retrospectively uploading data is generally possible, as Imperial College London demonstrated when they recently managed to report a trial’s result more than sixteen years after its primary completion date (NCT00390949, 5943 days overdue). Because of a lack of political and public pressure, there is currently little incentive for UK universities to report clinical trial results on the US registry as well as on EU CTR. Additionally, the previous lack of a ClinicalTrials.gov equivalent to the EU Trials Tracker website, which has aided universities and other trial sponsors in identifying clinical trials on EU CTR missing results, may have hindered progress in summary results posting on the US registry. Finally, we note that although the International Committee of Medical Journal Editors (ICJME) requires the registration of clinical trials on a primary registry as a condition for publication in academic journals, it does not require tabular summary results of trials to be uploaded onto trial registries [17]. We recommend that the ICJME expands its current policy to require summary results to be uploaded onto a trial registry before a paper is considered for publication in an academic journal, thereby incentivising routine adherence to scientific best practices as set out by the WHO.

The validity of our findings has been assured by the rigorous manual validation of the tracking tool. Our findings and tracker can be used to analyse reporting compliance on ClinicalTrials.gov and allow all trial sponsors to identify missing trial results. Of note, poor results reporting is not a problem unique to UK universities and a global analysis of missing trial results on the US registry would be useful as a means to highlight the performance of particular countries or institutions to ensure WHO best practices on clinical trial transparency are adhered to and to help guide policy development.

A limitation of our current study is that we were unable to determine the extent of the possible overlap between the US and the EU clinical trial registry, which could explain the poor performance of UK universities on ClinicalTrials.gov. However, we strongly believe that through trial registration on the US registry, sponsors are committing to keeping their trial status up to date and uploading results in accordance with WHO requirements, even if they register a clinical trial on two registries at the same time. Thus, even if a trial has been reported elsewhere, for example in the academic literature or on another primary registry, this does not exempt trial sponsors from the ethical commitment to update the details of their trial on ClinicalTrials.gov as well.

## Conclusion

Many major universities and hospitals across the European Union are currently working to improve their clinical trial reporting on EU CTR. Our findings raise concerns that these efforts may apply only to the small minority of trials involving investigative medicinal products that are registered on EU CTR, which are subject to reporting requirements under the EU Clinical Trial Regulation. Our findings suggest that non-commercial trial sponsors are not yet adopting WHO best practices in reporting the results of trials that cannot be registered on EU CTR. Such trials greatly outnumber trials of investigative medical products, and can be of equal or greater scientific and clinical importance; across Europe, they are most commonly registered on ClinicalTrials.gov, but also on other WHO primary registries such as ISRCTN and DRKS. Governments, regulatory agencies and research funders worldwide should extend registry reporting requirements to all interventional clinical trials as per WHO best practices [18]. We hope that our novel US clinical trials tracking tool (clinical-trials-tracker.com) will drive trial sponsors worldwide to exceed narrow regulatory compliance and fully implement WHO best practices to improve the completeness and accuracy of the medical evidence base and accelerate medical progress.

The scientific community is currently under immense pressure not only to produce novel health technologies for COVID-19, but also to prove rapidly and rigorously that they are safe and effective. The scrutiny of the transparency of clinical trial results for COVID-19 related health technologies should apply across the health sector, with detailed, timely and accurate reporting of clinical trials becoming the norm in order to facilitate evidence-based medicine during the pandemic and beyond.

## Supplementary Information

**Additional file 1.**

**Additional file 2.**
