# Supplementary material for: Clinical trial reporting performance of thirty UK universities on ClinicalTrials.gov—evaluation of a new tracking tool for the US clinical trial registry
Source: Trials. 2021 Jun 1;22:375. doi: 10.1186/s13063-021-05330-5 (PMC8169390; doi:10.1186/s13063-021-05330-5)
Supplement: Supplementary file 1 — Additional file 1. [file 13063_2021_5330_MOESM1_ESM.docx]

**Tracker Design and Manual Validation**

## Tracker Output (2.0)

### The tracker has different output categories for clinical trials, which are listed below. Figure 1 displays the process of how interventional clinical trials from ClinicalTrials.gov are sorted to these categories. The definitions of trial status as listed on ClinicalTrials.gov can be found [here](https://clinicaltrials.gov/ct2/help/glossary/recruitment-status). ‘Ongoing’ trials include the following statuses: “Active, not recruiting”; “Enrolling by invitation”; “Not yet recruiting” and “Recruiting”. The rate of due and reported trials from all due trials was calculated by using Formula 1 and categories 2 and 3.

$$Reporting Rate=\frac{Results and due}{\sum Due trials}$$

*Formula 1*

******

***Output Categories***

1. **No reporting requirement**
   All trials that are listed as ’Suspended’ or ’Withdrawn’ are not required to report results. They are sorted by the tracker to this category even if there is inconsistent data (e.g. no primary completion date) or if they have reported results (these results are ignored).
2. **Due but not reported**    
   Trials that have finished (’Completed’ or ’Terminated’) that have not posted results yet and have exceeded the reporting threshold. Trials that have finished that have not posted results and have no primary completion dates also will be classed under this category.
3. **Due and reported**    
   Trials that have finished (’Completed’ or ’Terminated’) that have reported their results. Late-posting of results will also be in this category.
   1. **Due and late reported**

Trials that have finished (’Completed’ or ’Terminated’) but have reported their results 395 days after their primary completion date.

- 1. **Due and reported in time**

Trials that have finished (’Completed’ or ’Terminated’) and have reported their results in time (within 395 days).    

1. **Completed/terminated but not due**
   1. Results not due yet
      Trials that have finished (’Completed’ or ’Terminated’) that have not posted results yet and are within the reporting threshold of 395 days.
   2. Results not yet expected but have reported
      Trials that have finished (’Completed’ or ’Terminated’) that are within the reporting threshold but have reported results.
2. **Ongoing**
   Trials that are ongoing with primary completion date in the future and have not posted results.
3. **Inconsistent data**    
   Trials that are:
   - inconsistent (’Unknown status’);
     - self-declared to be inconsistent.
   - ongoing trials that have completion date in the past.
     - These are trials that should have already been completed but are self-declaring as ongoing, this is contradictory and therefore inconsistent.
   - ongoing trials that have no termination date.
     - trials should have an expected primary completion date. Not having it is an inconsistency.

**Manual validation**

A random sample set of 10 U.K. universities were chosen to manually validate the tracker:

1. University of Oxford
2. University of Southampton
3. University of Leeds
4. King's College London
5. University of Edinburgh
6. Queen Mary University of London
7. University of Nottingham
8. London School of Hygiene and Tropical Medicine
9. University of Aberdeen
10. Imperial College London

The clinical tracker categories of inconsistent data, due trials with results and without and their resulting rate [Formula 1] were checked by comparing it with manual results. The tracker results were from data downloaded on 2^nd^ October 2020. Hence all trials with a primary completion date earlier than the 03.09.2019 (395 days prior) were considered due. For the manual validation, the data – excluding trials posted after the 2^nd^ October 2020 - was downloaded per university in a csv-format. The trials were then filtered and counted in an excel pivot table, the structure of the process is visualized in figure 2 (fig.2). The trials were categorized sequentially by Status, Primary Completion Date, and Results Availability. Trials with an ongoing status and a lacking or past primary completion date (tolerance of 30 days) and with the label unknown status were considered as inconsistent. The reporting rate of all overdue trials was calculated by dividing all due trials with reported results with the sum of due trials with and without results.

*Formula 1*

https://clinicaltrials.gov/ct2/results/refine -> Search details for Advanced Research:

**Study Type** = Interventional Studies (Clinical Trial) AND **Sponsors Lead** =**University’s name** ("Exact Match “) AND **first posted** before X

**Step-by-Step Guide for Manual Validation per University**^[[1]](#footnote-1)^

1. Download results with the US clinical-trials-tracker, date of download = date X
   1. The US clinical-trials-tracker will be found here: clinical-trials-tracker.com
   2. Until the website is finalised, the github code can be downloaded and used here: <https://github.com/LeeSean96/GlobalHealthRanking>
2. Go to <https://clinicaltrials.gov/ct2/results/refine> and define search criteria
   1. **Study Type** = Interventional Studies (Clinical Trial)
   2. **Sponsors Lead** =**University’s name** ("Exact Match “)
   3. **First posted** before date X
3. Choose following columns and download all data in a csv format:
   1. **status**
   2. **primary completion date**
4. Open excel, import data as csv and load it -> it will be a pivot table
5. Filter by column and fill in the count in the table**:**
   1. Filter by **status** for …:
      1. **ongoing**^[[2]](#footnote-2)^
         1. filter by **primary completion date** and **status** for:
            1. date before **30 days before** **date X or equals blank (exclude unknown status) ->** fill in category *1*
            2. **unknown status ->** add count to category *1*
      2. **completed or terminated**
         1. filter by **primary completion date** for:
            1. date before **395 days before** **date X or equals blank:**

filter by **results** for:

**no results** -> fill in category *2 (C2)*

**results** AND **results first posted** before **date X** -> fill in category *3 (C3)*

1. Calculate reporting rate by using values in *2* and *3* and *Formula 1*
2. Compare data to tracker results

$$Reporting Rate=\frac{Results and due}{\sum Duetrials}=\frac{C3}{C2+C3}$$

*Formula 1*

***Table 1:*** *manual validation results for the tracker*

| **Inconsistent** | *1* |
| --- | --- |
| **due but not reported** | *2* |
| **due and reported** | *3* |

**Manual validation time delay**

The mean and median time delay for results posting was also calculated for the random sample of ten universities. We did this by subtracting the results posting date from the primary completion date, which gave the number of days. We then calculated the mean and median per university from that.

***Figure 2: manual validation method for the tracker version****: via excel, dark blue coloring = validated*

*date of data download for U.S.-clinical-tracker = X, 395 days before X=X_due_, 30 days before X=X_threshold_* , *this step will be integrated in an updated version soon

Excel:

*csv file table with following columns:* **Study results, status, primary completion date**

Filter by **STATUS**

*Withdrawn or suspended*

1. **No reporting requirement**

Ongoing or Unknown

Filter by **PRIMARY COMPLETION DATE (PCD)** and **STATUS**

Completed or terminated

*PCD before X_threshold_ or blank OR status = unknown****:***

**6. *Inconsistent data***

Filter by **PRIMARY COMPLETION DATE(PCD)**

*PCD before/ equal X_due_*

*🡪*  Filter by **RESULTS**

*PCD after X_due_***:**

**4. completed/ terminated but not due**

*PCD after X_threshold_ AND status ≠ unknown***:**

**5. ongoing**

*No results reported*

*🡪* **2. due but not reported**

*Results reported AND Results first posted* before X *🡪* **3. due and reported**

*Calculate* difference between PCD and Results first posted (RFP) = D_RFP-PCD_*

*D_RFP-PCD_ < 395 days:*

***3b). due and reported in time***

*🡪* **3a). due but not reported**

*D_RFP-PCD_ ≥ 395 days:*

***3a). due and late reported***

*🡪* **3a). due but not reported**

1. It is recommended to do the manual validation for U.S.-clinical-tracker-data as soon as possible afterwards, since status will be changed of trials over time, which can falsify your results and you can not filter for time of status change. [↑](#footnote-ref-1)
2. Ongoing = [recruiting; unknown status; not yet recruiting; active, not recruiting; enrolling by invitation] [↑](#footnote-ref-2)
